# Study of the Economic, Environmental, and Social Factors Affecting Chinese Residents' Health Based on Machine Learning

**DOI:** 10.3389/fpubh.2022.896635

**Published:** 2022-06-14

**Authors:** Hui Xu, Wei Pan, Meng Xin, Wulin Pan, Cheng Hu, Dai Wanqiang, Ge Huang

**Affiliations:** ^1^Dong Fureng Institute of Economic and Social Development, Wuhan University, Wuhan, China; ^2^School of Applied Economics, Renmin University of China, Beijing, China; ^3^School of Economics and Management, North China Electric Power University, Beijing, China; ^4^School of Economic and Management, Wuhan University, Wuhan, China

**Keywords:** residents' health, economic factors, environmental factors, social factors, machine learning

## Abstract

The Healthy China Strategy puts realistic demands for residents' health levels, but the reality is that various factors can affect health. In order to clarify which factors have a great impact on residents' health, based on China's provincial panel data from 2011 to 2018, this paper selects 17 characteristic variables from the three levels of economy, environment, and society and uses the XG boost algorithm and Random forest algorithm based on recursive feature elimination to determine the influencing variables. The results show that at the economic level, the number of industrial enterprises above designated size, industrial added value, population density, and per capita GDP have a greater impact on the health of residents. At the environmental level, coal consumption, energy consumption, total wastewater discharge, and solid waste discharge have a greater impact on the health level of residents. Therefore, the Chinese government should formulate targeted measures at both economic and environmental levels, which is of great significance to realizing the Healthy China strategy.

## Introduction

Health is not only an inevitable requirement for promoting the overall development of society, but also a basic condition for improving economic productivity and efficiency (1). On July 15, 2019, the State Council of China issued the *Opinions of the State Council on Implementing the Healthy China Action*. It emphasized the establishment of a Healthy China Action Promotion Committee to formulate and distribute the Healthy China Action (2019–2030). The action estimated that by 2022, China will establish a health promotion policy system covering all areas of the economy and society. Then by 2030, China's national health literacy level and main health indicators of residents will improve and be consistent with that of high-level countries (2).

Although China's health industry has achieved long-term planning and development, industrialization, urbanization, and aging have brought changes in lifestyle and ecological environment (3). In recent years, the pressure and cost of health maintenance for residents in China have gradually increased because of infectious diseases like COVID-19 (4). At the end of 2020, the total number of medical and health institutions in China has reached 1,022,922, an increase of 1.53% over the previous year. The total health expenditure in 2020 is expected to reach 7,230.64 billion yuan, an increase of 9.83 percent over the previous year and accounting for 7.12 percent of GDP. Based on the above analysis, exploring the health influencing factors in China is of far-reaching significance for implementing the Healthy China strategy, improving the efficiency of health investment, and promoting the development and transformation of health service methods in China.

Health Ecology not only emphasizes the multi-layered nature of the individual and environmental factors which affect residents' health, but also highlights the complexity of these influencing factors (5). Firstly, personal characteristics such as gender, age, genetic factors have an impact on health. For example, the mother's age and education level affected infant mortality (6). Many diseases, such as cardiovascular disease, diabetes and malignant tumors, are related to bad living habits and sleeping habits (7–9). Secondly, personal social networks also significantly affect personal health. Researches have shown that spouse interaction (10), parents' social status and behavior (11, 12), and education level (13) may have an impact on health. In addition, external factors such as economy, politics, environmental pollution, local culture and education also impact personal health. The health of residents may be affected by socio-economic foundation and wealth distribution methods due to inequality in income levels (14, 15). Li and Zhang (16) believed that the public health expenditure and medical service actions could control the disease and then affect the health level of residents. There were also many studies showing that air pollution (17) and regional culture (18) impact the behavior and health of residents. Additionally, COVID-19 is more likely to occur in prosperous cities closer to the epicenter, located on higher altitudes, with high concentrations of air pollutants except NO_2_ and O_3_, under conditions of extreme weather and high minimum relative humidity (19). Zhao et al. (20) found the residents' environmental health literacy (EHL) level of urban residents is significantly higher than that of rural residents in Shaanxi Province, so they stressed the importance of education and popularizing basic environmental health knowledge.

From the above analysis, scholars have done a lot of research on the influencing factors of health, but there are still some limitations. Firstly, the existing studies focus on the analysis of single factors affecting health and underestimate the interactions between various factors. Secondly, the current analysis of health influencing factors is mainly based on logistic model (14), descriptive comparative analysis (21) and DEA model (22). However, many factors are not completely independent, so it is inappropriate to select relevant variables for direct regression analysis. Experts are increasingly seeing greater potential for using machine learning in a wider range of health efforts. Compared with traditional statistical methods, machine learning has the following advantages. Firstly, Machine learning is conducive to establish the multivariate empirical relationship between causes and effects (23). Secondly, system dynamics modeling approaches or agent-based models contributes to arrange and assess the weights of interventions at multiple levels (24, 25). Thirdly, by incorporating more information and handling missing data, machine learning can be used for resource allocation and result prediction (26).

Considering the deficiencies of previous studies, this paper firstly selects 17 characteristics from the economic, environmental and social levels to analyze the influencing factors of health, and innovatively adds the characteristics of carbon emission trading in the context of carbon neutrality. Secondly, instead of traditional analysis methods, this paper uses machine learning methods to filter the independent variables, and obtains the optimal model and fitting results. The rest of this paper is as follows: Data and methodology presents the machine learning method, explains the variable selection criteria and data sources. Empirical analysis and discussion analyzes the relevant results. Conclusions and policy implications summarizes the conclusions and puts forward corresponding policy suggestions.

## Data and Methodology

Practical problems often involve a large number of features, however not all of them are necessary, and many of them are redundant or even irrelevant, which may degrade the performance of models. Feature selection is an important technology of feature engineering. It can automatically remove repeated, redundant, and irrelevant features in the data, filter out the attributes most closely related to the target problem to construct feature subsets, thereby reducing the complexity of the model and enhancing the interpretability. Among them, the recursive feature elimination method can calculate the prediction effect of each subset in the feature space for a given learner, and select the feature subset that can promote the learner to achieve optimal performance. The advantage of this method is that it can maximize the performance of the learner and achieve a “tailor-made” effect. Therefore, this paper adopts the XG-boost algorithm and Random forest algorithm based on recursive feature elimination to select the key factors affecting health.

### XG-Boost

XG-boost is an improved Gradient Boosting Decision Tree (GBDT) algorithm (27). Using the addition model, GBDT reduces the residuals generated in the training process to achieve data classification or regression. Through multiple iterations, each iteration produces a weak classifier based on the residuals of the previous classifiers. Generally, the requirements for weak classifiers are simple enough, with low variance and high deviation. XG-boost is an efficient implementation of GBDT. Its advantages are mainly reflected in the following aspects:

1. XG-boost controls the complexity of the model by adding regular terms to the cost function. The regular term contains the number of leaf nodes of the decision tree and the sum of the squares of the L2 modules, which are output on each leaf node. From the perspective of bias and variance balance, the regular term reduces the complexity of the model, makes the learned model simpler, and prevents overfitting.
2. XG-boost performs a second-order Taylor expansion on the cost function and uses both the first-order and second-order derivatives, while the traditional GBDT only uses the first-order derivative information. From this point, XG-boost not only accelerates the convergence speed of the model, but also improves the accuracy of loss definition.

The objective function of XG-boost is:

(1) $$\begin{array}{r} L_{K}\left(F\left(x_{i}\right)\right)=\sum_{i=1}^{n}\Psi \left(y_{i},F_{K}\left(x_{i}\right)\right)+\sum_{k=1}^{K}\Omega \left(f_{k}\right) \end{array}$$

In the Equation (1), Ω(*f*) = γ*T* + 0.5λ ||ω||^2^. And γ is the complexity parameter, T is the number of leaf nodes, λ is the fixed coefficient, and ||ω||^2^ is the L2 norm of the leaf weight. The first part of the function represents the training loss of the model, and the second part represents the sum of the complexity of each tree.

The original objective function after Taylor expansion can be expressed as follows:

(2) $$\begin{array}{r} L_{K}≅\sum_{i=1}^{n}\left[g_{i}f_{k}\left(x_{i}\right)+\frac{1}{2}h_{i}{f_{k}}^{2}\left(x_{i}\right)\right]+\Omega \left(f_{k}\right) \end{array}$$

In the Equation (2), *g*_*i*_ = ∂_*F*_*k*−1_(*x*_*i*_)_Ψ(*y, F*_*k*−1_(*x*_*i*_)) represents the first order of the loss function and $h_{i}=\partial_{F_{k-1}\left(x_{i}\right)}^{2}\text{Ψ}\left(y,F_{k-1}\left(x_{i}\right)\right)$ represents two-step statistics.

XG-boost also adds algorithms for sparse data. For samples lacking eigenvalues, XG-boost can learn its splitting direction autonomously. It uses a greedy search algorithm to find the best tree structure. Suppose *I*_*L*_ and *I*_*R*_ represent the sample sets of the left and right branches, respectively. For each node on the tree, XG-boost tries to add a split. The benefits of the split can be expressed in the following equation:

(3) $$\begin{array}{r} G=\frac{1}{2}\left[\frac{{\left(\sum_{i\epsilon I_{L}}g_{i}\right)}^{2}}{\sum_{i\epsilon I_{L}}h_{i}+\lambda }+\frac{{\left(\sum_{i\epsilon I_{R}}g_{i}\right)}^{2}}{\sum_{i\epsilon I_{R}}h_{i}+\lambda }-\frac{{\left(\sum_{i\epsilon I}g_{i}\right)}^{2}}{\sum_{i\epsilon I}h_{i}+\lambda }\right]-\gamma \end{array}$$

### Random Forest

The Random forest model uses a random method to build a forest. The forest contains many decision trees, which are weakly correlated or even irrelevant, but with high prediction accuracy. Based on the classification results of these decision trees, the principle of minority obedience to the majority is adopted to make a comprehensive integration judgment (28).

The randomness of random forest is reflected in two aspects: one is the randomness of sample selection; The second is the randomness of feature selection. Through the perturbation of samples and features, the variance of the classifier is reduced faster, and the ability to deal with unbalanced data is improved. The flexible and practical random forest algorithm can run effectively on large data sets, process input samples with high-dimensional features, and rank the importance of features as an embedded feature selection method. Its learning process is fast and has good statistical robustness. The specific steps of Random forest are as follows:

1. The method randomly selects a certain number of samples from the training set as the root node samples of each tree.
2. When building the decision tree, the model randomly selects several candidate features and selects the most suitable feature as the split node.
3. After the Random forest is established, the model enters each decision tree for type output or regression output for the test sample. If it is a classification problem, the final category is output by voting. If it is a regression problem, the average value of each decision tree output is the final result.

Classification and Regression Tree (CART) is constructed by the principle of least mean square deviation method for regression problems. In other words, for any divided feature A, the method divides both sides of the point S arbitrarily, named the data sets *D*_1_ and *D*_2_. And then, the model finds the feature and eigenvalue dividing points corresponding to the minimum mean square error of each set of *D*_1_ and *D*_2_ and the minimum sum of the mean square error of *D*_1_ and *D*_2_. The expression is:

(4) $$\begin{array}{r} \underset{A,s}{\underset{︸}{min}}\left[\underset{c_{1}}{\underset{︸}{min}}\underset{x_{i}\notin D_{1}\left(A,s\right)}{\sum }{\left(y_{i}-c_{1}\right)}^{2}+\underset{c_{2}}{\underset{︸}{min}}\underset{x_{i}\notin D_{2}\left(A,s\right)}{\sum }{\left(y_{i}-c_{2}\right)}^{2}\right] \end{array}$$

In Equation (4), *c*_1_ is the sample output average of the *D*_1_ data set, and *c*_2_ is the sample output average of the *D*_2_ data set.

The prediction of CART is based on the mean value of the leaf nodes. Therefore, the prediction of random forest is the average of the predicted values of all trees. In addition, the random forest can also evaluate the importance of features, mainly measured by the error rate of out-of-bag data (OOB). The formula is as follows:

(5) $$\begin{array}{r} MDA(A_{i}\text{)=}\frac{1}{ntree}\overset{ntree}{\underset{t=1}{\sum }}\left(errOOB_{\text{t}1}-errOOB_{\text{t2}}\right) \end{array}$$

In Equation (5), *ntree* represents the number of times the feature *A*_*i*_ appears in the forest, *errOOB*_*t*1_ represents the out-of-bag error after the *A*_*i*_ attribute value changes in the t-th tree, and *errOOB*_*t*2_ represents the out-of-bag error of the normal *A*_*i*_ value in the t-th tree.

### Variable Selection

#### Selection of Health Variables

At present, scholars have great differences in measuring residents' health levels, and there is no unified measurement standard (29). In the *Healthy China 2030* plan, population mortality, average life expectancy, neonatal mortality, maternal mortality, mortality of children under 5 years old, and the proportion of urban and rural residents meeting the *National Fitness Standards* and above are defined as the measurement of health level. Among them, population mortality and average life expectancy can more comprehensively reflect the overall health status of the region (30). Therefore, considering the completeness and availability of data, this article selects population mortality as an indicator to measure the health level of residents.

#### Selection of Characteristic Variables

In addition to innate genetic factors, health is also largely affected by external environmental factors such as social and economic foundations, wealth distribution methods, medical conditions, health resource allocation, and living and working environments. An individual's age, gender, and socioeconomic status can also affect health status. Besides, health awareness, education level, and lifestyle are also important influencing factors. Therefore, the health factors can be summarized from three aspects: economy, environment, and society.

From the perspective of economic factors, health issues are closely related to the economic development of a country or a region. Grossman regards health as an important aspect of human capital (31). At present, China is in a critical period of economic transformation and upgrading, and further economic growth requires a higher and higher quality of human capital. From the perspective of environmental factors, environmental pollution brought by economic development has seriously threatened human health. The World Health Organization (WHO) believes that environmental pollution is the biggest threat to human health. Epidemiology has also confirmed the relationship between health problems such as tumors, respiratory diseases, cardiovascular and cerebrovascular diseases, and environmental pollution (32–34). From the perspective of social factors, the length of education and education funding are also closely related to the health of residents. Therefore, referring to the summary of economic, environmental, and social factors in the existing literature and combined with China's actual national conditions, the characteristic variables selected in this paper are shown in Table 1. Table 2 shows the classification of characteristic variables.

**Table 1:** Corresponding codes of characteristic variables.

**Feature variables**	**Code**
Imports and exports as a percentage of GDP	x1
The level of urbanization	x2
Number of industrial enterprises above the scale	x3
Industrial value added	x4
Population density	x5
Carbon trading rights	x6
Coal consumption	x7
Energy consumption	x8
Annual per capita health expenditure	x9
The number of years of education per capita	x10
Ratio of the number of higher education	x11
Total wastewater discharge	x12
Total SO_2_ emissions	x13
Total NO emissions	x14
Total particulate emissions	x15
Total solid waste emissions	x16
GDP per capita	x17

*Extract and integrate literature and data related to health problems*.

**Table 2:** Classification of characteristic variables.

**Classification**	**Feature variables**
Economic dimension	x1, x2, x3, x4, x5, x17
Environmental dimension	x6, x7, x8, x12, x13, x14, x15, x16
Social dimension	x9, x10, x11

*Extract and integrate literature and data related to health problems*.

### Data Sources

The carbon emissions permit trading started with the *Notice on the Pilot Work of Carbon Emission Trading* issued by China in 2011. Given the availability of data, this paper selects the inter-provincial panel data of 31 provinces, municipalities, and autonomous regions in China from 2011 to 2018 as the sample. Among them, the population mortality rate, per capita GDP, urbanization level, the proportion of total imports and exports in GDP, years of education per capita, and the proportion of population with higher education are all derived from the *China Statistical Yearbook* over the years; total wastewater discharge, total SO_2_ emissions, total NO emissions, total particulate matter emissions, and total solid waste emissions are all derived from the *China Energy Statistical Yearbook* over the years; coal consumption and energy consumption are derived from the *China Environmental Statistical Yearbook* over the years; industrial enterprises above designated size and industrial added value are derived from the National Bureau of Statistics^1^; the population density is derived from the WIND^2^ database; the annual per capita health expenditure is derived from the *China Health Statistics Yearbook*.

## Empirical Analysis and Discussion

### XG-Boost

#### Parameter Selection in XG-Boost

When using machine learning algorithms, the parameters of the algorithm are very important. Parameters can not only make the prediction of the model more accurate, but also make the training effect of the model better. Therefore, it is necessary to adjust the parameters before modeling. The parameters of the XG-boost algorithm are mainly divided into three types, namely General Parameters, Booster Parameters, and Learning Task Parameters. When modeling with the XG-boost algorithm, the function uses the default parameters. In order to pursue better results, this article tunes the parameters before modeling. After determining the optimal parameters, this paper keeps them unchanged to ensure a fair comparison between the models and analyze the weights of features under the same parameters.

The parameter *max_depth* represents the maximum depth of the tree. If the depth of the tree is too large, the model would overfit. This article sets *max_depth* to 7. *n_estimators* represents the number of decision trees in the model. To determine the optimal *n_estimators* value, this model makes *n_estimators* equal to 200. When *n_estimators* is 100, the error value of the model tends to be stable. The parameter *eta* represents the learning step length, and the general value range is [0, 1]. If the learning rate is too high, it is easy to overfit, so this paper chooses *eta* equal to 0.1. Other detailed parameters are shown in Table 3.

**Table 3:** XG-boost parameter values.

**XGB experimental parameters**	**Value**
gamma	5
max_depth	7
subsample	0.5
eta	0.1
nthread	−1
num_round	100

*Parameter tuning during XGBoost model building*.

#### Importance Ranking of Variables in XG-Boost

XG-boost filters variables through variable importance scores. Generally speaking, the importance score measures the value of the feature in constructing the improved decision tree. In a single decision tree, the attribute importance is calculated by improving the performance metric of each attribute split point, and the node is responsible for weighing and recording times. In other words, the greater the improved performance of an attribute on the split point, the closer to the root node, the greater the weight, and the more boosted trees are selected, the more important the attribute is. Finally, the weighted sum of the results of an attribute in all boosted trees is then averaged to obtain the importance score.

**Figure 1:**
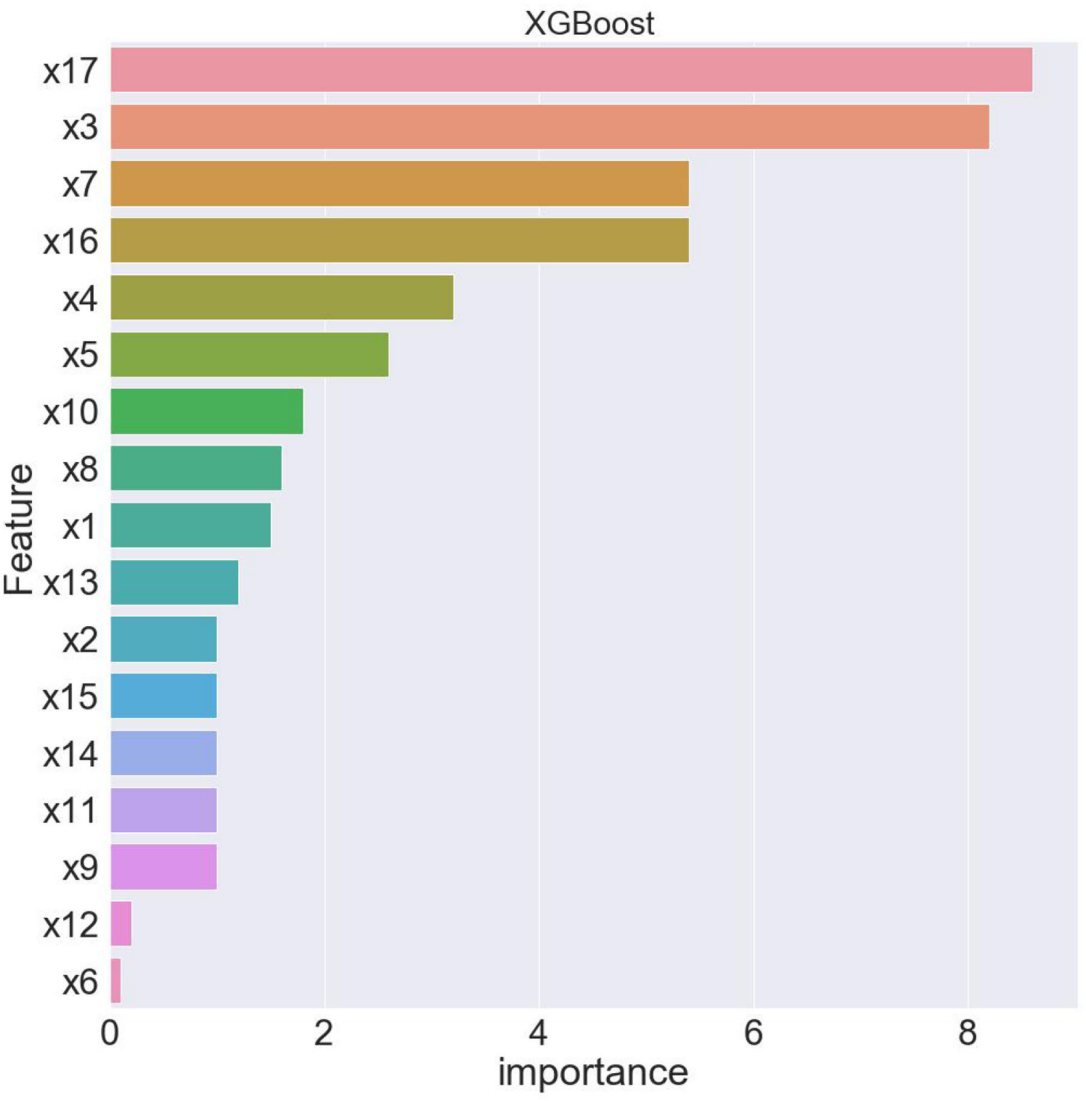
Feature ranking of XG-boost.

The XG-boost algorithm can generate three types of variable importance indicators: variable importance ranking, importance score and weight value. In this paper, the importance of each characteristic variable to the health level of residents is ranked based on the variable importance scores. The result is shown in Figure 1. The horizontal axis of Figure 1 represents the importance of variable scores, and the vertical axis variables are arranged in descending order of importance.

In order to get the optimal model, this article uses the forward selection method to determine the relatively important feature variables, so that the model has the strongest degree of interpretation. At the same time, to ensure the stability of the experimental results, the five-fold cross-validation method is used to evaluate the predictive ability and robustness of the model. First, the samples are divided into training samples and test samples. The principle is to randomly divide the samples into five groups, four of which are used as training samples to build the model, and then use the remaining group as test samples to test the effect of the model. After that, the samples are replaced, and the test samples are tested repeatedly. The final results of the five-fold crossover experiment are shown in Table 4.

**Table 4:** XG-boost's five-fold crossover experiment results.

**Sample**	**Evaluation indicators**	**All features (21)**	**x17, x3, x16, x7, x4**	**x17, x3, x16, x7, x4, x5**	**x17, x3, x16, x7, x4, x5, x10**	**x17, x3, x16, x7, x4, x5, x10, x8**
Training samples (80%)	Root mean square error (RMSE)	0.41944	0.420202	0.426395	0.426798	0.424173
Test samples (20%)	RMSE	0.57756	0.573808	0.5055197	0.516337	0.514387

*Output of fitting results of XGBoost model in this study*.

**Figure 2:**
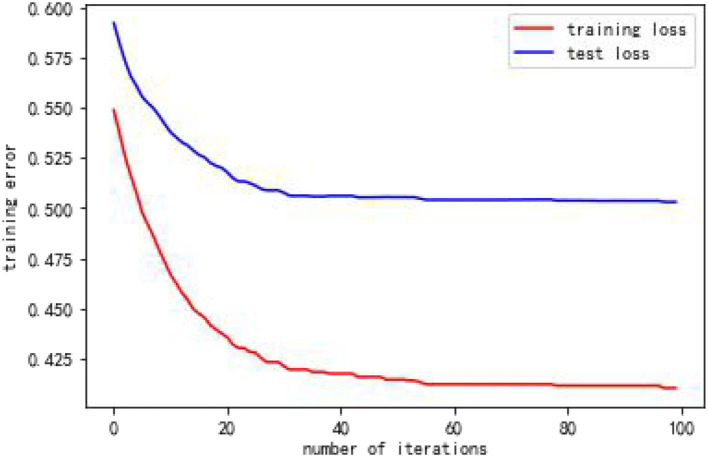
Error variation of XGBoost model over 100 iterations.

**Figure 3:**
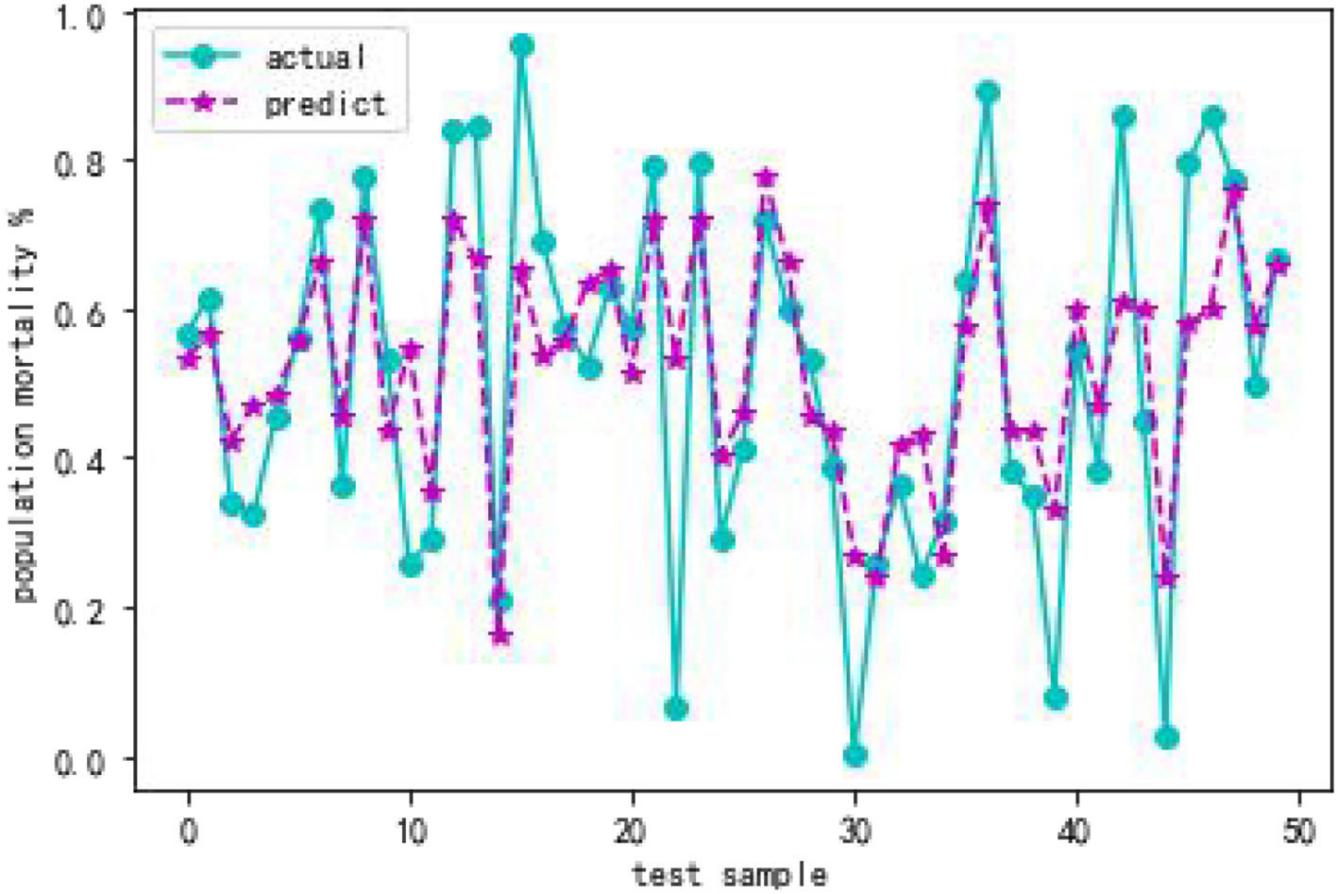
Comparison of XGBoost model predicted value and actual value.

From Table 4, when using full range features for analysis, the root mean square errors for the training and test samples was 0.41944 and 0.57756, respectively. Taking all feature modeling analysis as the baseline, when only the top five important feature variables are selected for training models, the root mean square errors of the training samples and test samples are 0.420202 and 0.573808, respectively. When the feature x5 is added according to the importance, the root mean square errors of the training sample and the test sample become 0.426395 and 0.5055197, respectively, and the effect is obviously better. When features x10 and x8 are added continually, the effect rebounds, and the performance of the model is unsatisfactory. Based on the above analysis, this article finally selects the six features of x17, x3, x16, x7, x4, and x5. Figures 2, 3 are performance descriptions of the XG-Boost model built on these six features. It can be seen from Figure 2 that with the increase of the number of iterations, the errors of the model in both the training set and the test set are continuously reduced, and finally tend to be stable. Figure 3 compares the population mortality predicted by the test set model with the true population mortality, and the difference between the two is acceptable in most samples.

### Random Forest

#### Parameter Selection in Random Forest

The two most important parameters of the random forest model are the number of variables selected by the node branches of the decision tree and the number of decision trees. In order to pursue better results, this article tunes the parameters before modeling. Similarly, after determining the optimal parameters, this paper does not adjust them to ensure a fair comparison between the model constructed using some features and the full feature model, and to ensure the importance of features is analyzed under the same parameters.

The value of *max_depth* is [1, 6]. Excessive *max_depth* will cause the model to overfit. Here the value of *max_depth* is set to 3. When *n_estimators* is 200, the error value of the model tends to be stable. Other detailed parameters are shown in Table 5.

**Table 5:** Random forest parameter values.

**Random forest experimental parameters**	**Value**
n_estimators	100
max_depth	3
random_state	10
min_samples_split	2
min_samples_leaf	1
criterion:	“mse”

*Parameter tuning during Random forest model building*.

#### Importance Ranking of Variables in Random Forest

The random forest algorithm uses OOB error to calculate and rank feature variables' relative importance. When many features participate in classification, this method is very suitable because the high correlation between many features will cause high-dimensional problems, significantly reducing extraction accuracy. At this stage, the feature space of machine learning models is often huge and complex, showing complex characteristics such as high-dimensionality and non-linearity. For such massive high-dimensional data, eliminating redundant features for feature screening has become one of the important issues faced by information technology today.

The basic idea and process of random forest algorithm for ranking the importance of feature variables are as follows:

1. For each variable, the out-of-bag data error corresponding to each tree is calculated and recorded as Err_OOB1_. The probability that each sample is not drawn is *(1-1/N)N*. When *N* is large enough, *(1-1/N)N* will converge to 1/e≈0.368. That is, nearly 37% of the samples will not be drawn (35).
2. Noise interference is added to the variables of the data outside the bag. That is, the sequence is changed randomly, and the error of the data outside the bag is calculated again, which is recorded as Err_OOB2_. The importance of a variable can be estimated by analyzing the increase in the error of the data outside the bag when the data sequence changes outside the bag. Suppose the importance of the variable is *M* and the number of decision trees is *n*_*tree*_, *M* = ∑(*Err*_*OOB*2_ − *Err*_*OOB*1_)/*n*_*tree*_. *M* can explain the importance of variables, because the accuracy of out-of-bag data drops significantly after adding random noise. That is, Err_OOB2_ rises. This shows that this variable has a great influence on the prediction results of the sample. In other words, the degree of importance is relatively high.

**Figure 4:**
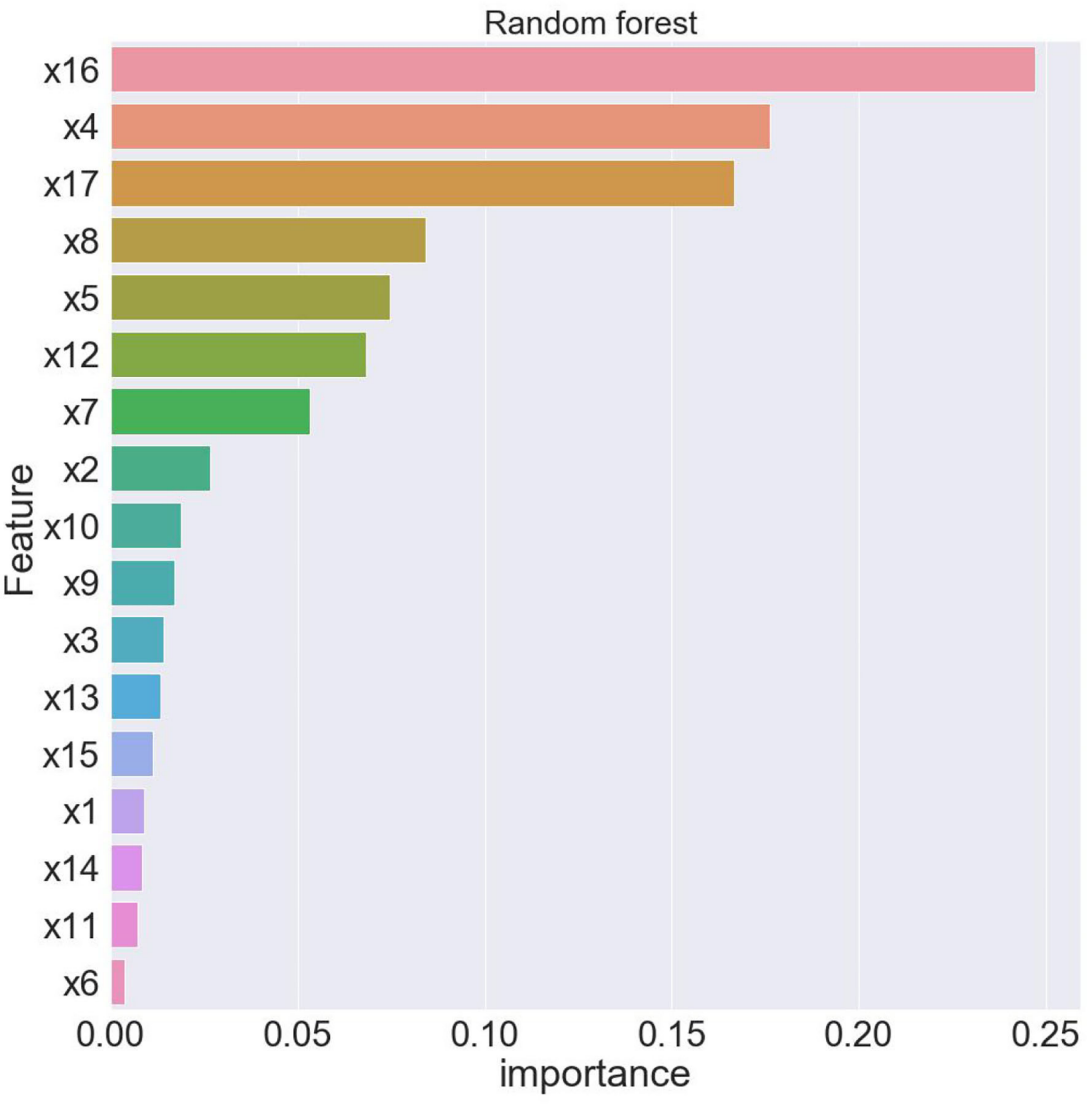
Feature ranking of random forest.

As shown in Figure 4, this paper uses the random forest algorithm to rank the importance of features that affect the health of Chinese residents. The horizontal axis of Figure 4 represents the variable importance scores, and the vertical axis variables are arranged in descending order of importance.

In order to obtain the optimal model and ensure the stability of the experimental results, this paper uses the five-fold cross-validation method to evaluate the predictive ability and robustness of the model. The results of the five-fold crossover experiment are shown in Table 6.

**Table 6:** Random forest's five-fold crossover experiment results.

**Sample**	**Evaluation indicators**	**All features (21)**	**x16, x4, x17, x8**	**x16, x4, x17, x8, x5**	**x16, x4, x17, x8, x5, x12**	**x16, x4, x17, x8, x5, x12, x7**
Training samples (80%)	RMSE	0.464821	0.491444	0.481829	0.4803921	0.470389
Test samples (20%)	RMSE	0.536169	0.563798	0.549059	0.542879	0.535904

*Output of fitting results of Random forest model in this study*.

**Figure 5:**
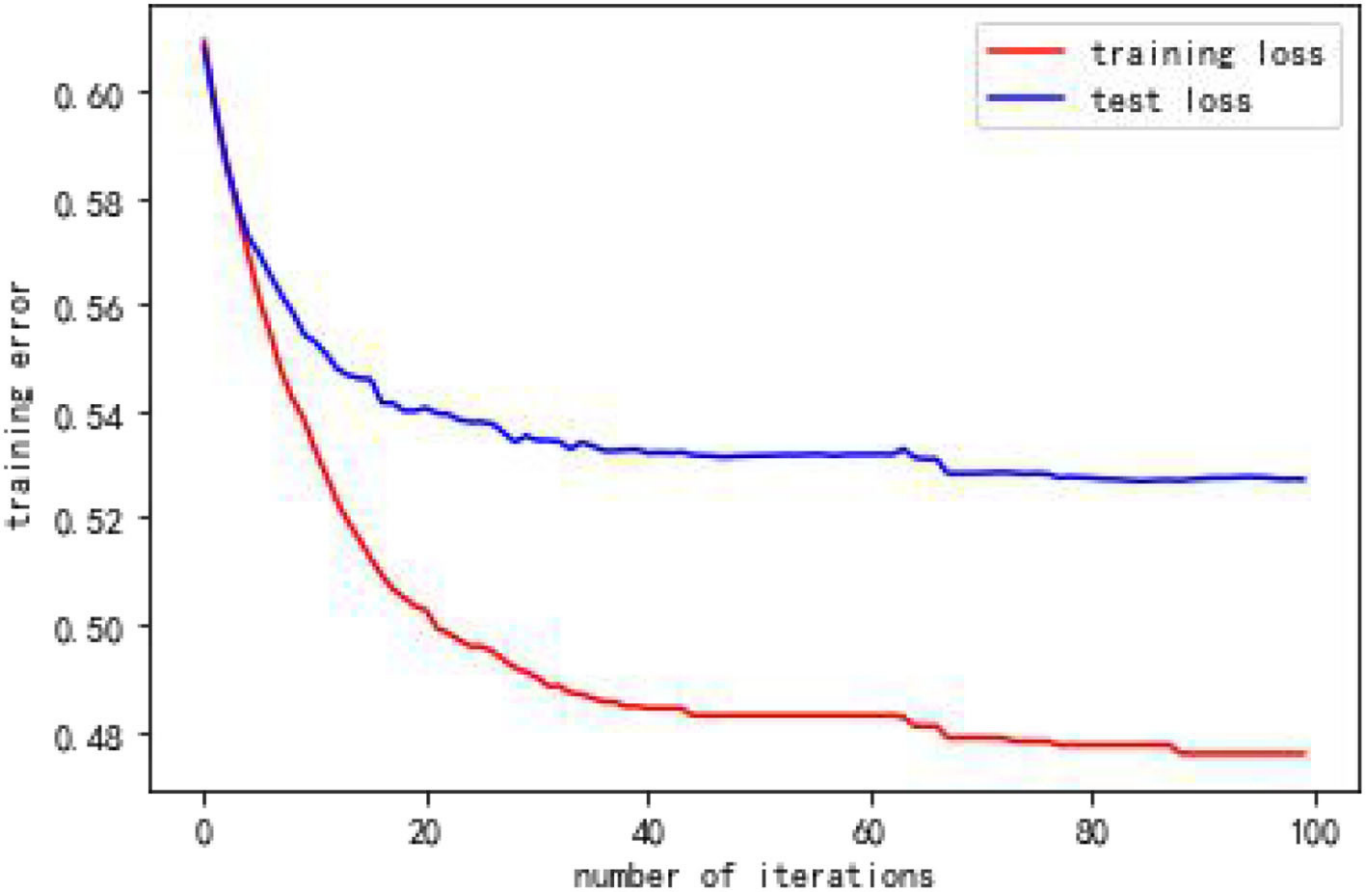
Error variation of random forest model over 100 iterations.

**Figure 6:**
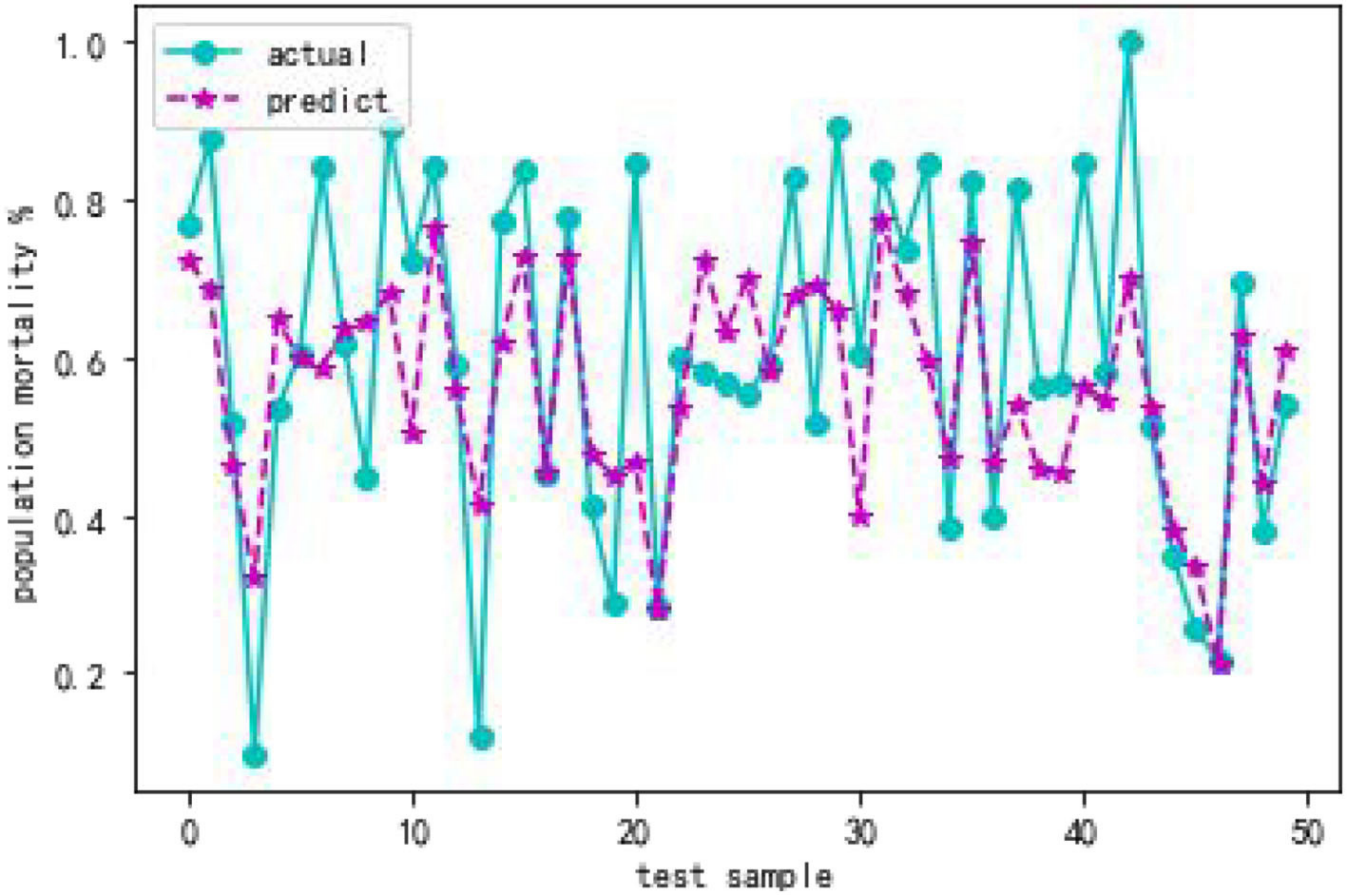
Comparison of random forest model predicted value and actual value.

From Table 6, when using full range features for analysis, the root mean square errorsof training and test samples was 0.464821 and 0.536169, respectively. Take all feature modeling and analysis as the baseline, and use different feature numbers for comparison and analysis. When only the top four important feature variables are selected to train the model, the root mean square errors of the training samples and test samples are 0.491444 and 0.563798, respectively. The results of the root mean square error corresponding to all features models are quite different. This shows that only four features are not enough to support the prediction of the whole model, and the loss of information is relatively large. In the same way, until the analysis of the first seven feature variables, there is a small gap between the root mean square error of training samples and test samples and the root mean square error corresponding to the full feature. Therefore, this article finally chooses seven features of x16, x4, x17, x8, x5, x12, x7. Figures 5, 6 depict the predictive effect of the random forest model on population mortality. The model stabilizes after about 50 iterations, and the prediction error in the test set is also in an acceptable range.

### Feature Importance Analysis

From the above analysis, we can use the XG-boost algorithm to screen out the six characteristic variables of x17, x3, x16, x7, x4 and x5, which have a relatively high impact on the health level of residents. Using the Random forest algorithm, we screened out the seven characteristic variables, x16, x4, x17, x8, x5, x12 and x7. In order to better grasp the influencing factors of Chinese residents' health level from a macro perspective, this paper combines the characteristic variables selected by the two methods. Finally, eight characteristic variables of x3, x4, x5, x7, x8, x12, x16, x17 are determined. That is, among economic factors, the number of industrial enterprises above designated size, industrial added value, population density, and per capita GDP have a greater impact on the health of residents. Among the environmental factors, coal consumption, energy consumption, total wastewater discharge and solid waste discharge have a greater impact on the health level of residents.

1. Among economic factors, the number of industrial enterprises above designated size, industrial added value, and per capita GDP represents the economic development level of a region (36). On the one hand, the rapid development of industry has promoted the improvement of China's economic level. The improvement of the national economic level is conducive to increasing health investment and promoting the development of medical and health care, which creates better material conditions for preventing, controlling, and eliminating certain diseases (37). On the other hand, the process of industrialization has caused a large amount of vegetation damage. The rapid economic development also increases the discharge of waste, waste gas and wastewater, all of which have brought serious harm to the health of residents. Therefore, the number of industrial enterprises above designated size, industrial added value, and per capita GDP have a greater impact on the health of residents.
  The population density reflects the spatial agglomeration characteristics of the population that is, the number of people per unit land area in different regions (38). The increase in population density promotes the coverage of health resources to a larger population, effectively avoids the waste of medical resources, and promotes the effective utilization of medical resources and the health output of residents. But at the same time, the high residential population density also brings a large amount of traffic flow and automobile exhaust emissions, which indirectly causes air pollution and further increases the risk of illness. Even if there is no significant change in air quality, the growth and migration of the urban population lead to an increase in the number of people exposed to air pollution. High exposure brings about changes in the health of residents (39). Therefore, population density has a greater impact on the health of residents.
2. Among environmental factors, coal consumption and energy consumption are general indicators that reflect the level of energy consumption. Energy consumption has a dual impact. On the one hand, energy plays a key role in social and economic development. On the other hand, it has brought serious environmental pollution problems (40). In particular, the primary energy consumption and emissions based on fossil fuels such as coal have released various air pollution, such as CO, SO_2_, soot particles, and PM_2.5_. Air pollution can cause and aggravate various diseases, such as respiratory diseases and lung cancer. Therefore, coal consumption and energy consumption have a more significant impact on the health of residents.

The total discharge of wastewater and solid waste represent the discharge of pollutants. The problem of water pollution and solid waste has always been one of the important factors restricting the economic development of many regions in China. At the same time, it seriously affects public health and social welfare. Wastewater contains a large number of pathogenic microorganisms, many of which can spread through water and cause various diseases. Wastewater also contains various types of heavy metal pollutants, which are carcinogenic, mutagenic and teratogenic to the human body (41). Solid waste can cause serious harm to water, atmosphere and soil. If water, the atmosphere, and the soil are all polluted, the pollutants can enter the human body through the respiratory tract and digestive tract, causing serious effects on human health. Therefore, the total amount of wastewater discharge and solid waste discharge have a greater impact on the health of residents.

## Conclusions and Policy Implications

Although the existing studies have comprehensively analyzed the factors affecting the health level of residents, considering the impact of only one factor may underestimate the interaction or combination of various factors. Therefore, based on China's provincial panel data from 2011 to 2018, this paper selects 17 characteristic variables from three levels of economy, environment and society, and uses the XG-boost algorithm and random forest algorithm based on recursive feature elimination to select the variables. The results show that at the economic level, the number of industrial enterprises above designated size, industrial added value, population density and per capita GDP have a greater impact on the health of residents; At the environmental level, coal consumption, energy consumption, total wastewater discharge and solid waste discharge have a greater impact on the health level of residents.

Based on the above conclusions, this article puts forward the following suggestions:

1. At the economic level, we should continue to promote the development of industrial green and low-carbon recycling firstly. In 2020, China proposed to achieve a carbon peak by 2030 and achieve carbon neutrality by 2060. The industry is an important area of carbon emissions, accounting for about 70% of emissions. Therefore, to achieve the dual-carbon goal, industrial enterprises should accelerate green transformation, change their development methods, and continuously improve the quality contribution of green total factor productivity to industrial economic growth (42). On the one hand, we should vigorously develop strategic emerging industries and high-tech industries. On the other hand, we should accelerate the elimination of outdated production capacity, rationally allocate scarce resources, constantly improve the quality of economic growth, and finally realize the green transformation of the industrial economy. The second is to reasonably adjust the population density. By optimizing the land use structure of the urban area and increasing land for public service facilities, green areas and squares, and road traffic, we promote the coordinated development of the residential and employment populations.
2. At the environmental level, it is necessary to accelerate energy transformation and energy technology innovation, so as to reduce the damage of pollutants from energy consumption to residents' public health. For a long time, the Chinese energy consumption structure has been dominated by coal (43). Therefore, the coal-based energy structure needs to be changed. The Chinese government can increase the proportion of clean energy consumption through energy transformation, reduce the consumption of traditional energy such as coal as much as possible, and promote the development of energy production and consumption toward a cleaner, low-carbon, and high-efficiency direction. At the same time, we should increase investment in science and technology innovation and policy support, and promote the innovation, application and promotion of industrial low-carbon equipment. Reduce the discharge of CO, SO_2_, wastewater, solid waste, and other pollutants through technological innovation.

Although this article has deeply analyzed the influencing factors of residents' health level in China, there are still some shortcomings, which should be further improved in the following two aspects in the future. Firstly, this article does not divide the region to study the factors affecting the health level of residents, and the research area should be expanded in the future. Second, given the availability of data, this article uses a single indicator to measure the health of residents, and constructing a reasonable and objective health indicator system will be our future research direction.

## Data Availability Statement

The original contributions presented in the study are included in the article/supplementary material, further inquiries can be directed to the corresponding authors.

## Author Contributions

WeP and MX contributed to study design and wrote the manuscript. DW, HX, GH, WuP, and CH collected and analyzed the data. HX interpreted results. All authors have read and agreed to the published version of the manuscript.

## Funding

This research was funded by National Natural Science Foundation of China (NSFC) (Grant Nos. 71871169, U1933120, J2024015) and fund for building world-class universities (disciplines) of Renmin University of China Project No. KYGJC2021005.

## Conflict of Interest

The authors declare that the research was conducted in the absence of any commercial or financial relationships that could be construed as a potential conflict of interest.

## Publisher's Note

All claims expressed in this article are solely those of the authors and do not necessarily represent those of their affiliated organizations, or those of the publisher, the editors and the reviewers. Any product that may be evaluated in this article, or claim that may be made by its manufacturer, is not guaranteed or endorsed by the publisher.

## Footnotes

^1^ http://www.stats.gov.cn

^2^ https://www.wind.com.cn/newsite/edb.html
